# Personality, Intelligence, and Second Language Learning Success: A Systematic Review

**DOI:** 10.3390/bs15040428

**Published:** 2025-03-27

**Authors:** Wei Xu

**Affiliations:** Faculty of Humanities and Social Sciences, City University of Macau, Macau 999078, China; weixu@cityu.edu.mo

**Keywords:** personality, emotional intelligence, second language learning, individual differences, intelligent personal assistants

## Abstract

This paper discusses the interrelation between personality and intelligence in acquiring a second language (L2). From 13 studies and the available literature, it determines that extraversion, openness, and conscientiousness, as well as subdomains of emotional intelligence (well-being, empathy, and sociability) are important in predicting L2 motivation, perseverance, and achievement. The interpretation also outlines L2-specific trait emotional intelligence (TEI) and cultural intelligence as the key factors explaining why learning may be consistent with various measures other than general variables. Furthermore, it elaborates on how self-control, perseverance, and mindset assist in coping with language learning anxiety and promoting greater engagement with language learning. However, there are some limitations to this study, focusing on cross-sectional data and the homogeneity of the learner population. Therefore, follow-up work should take into account a longitudinal approach, examine the role of IPAs in learning L2, and move towards integrative perspectives in which the dispersive connections between traits of personality and intelligence and L2 proficiency would be adequately captured.

## 1. Introduction

Recent studies have conducted systematic reviews or meta-analyses of personality and intelligence (Anglim et al., 2022; Vashisht et al., 2021), personality and academic achievement (Meyer et al., 2023; Quílez-Robres et al., 2023), and personality and cognitive ability (Stanek & Ones, 2023); however, there have been no such studies examining the relationship between personality, intelligence, and second or foreign language (L2) learning. Individual differences—most notably, personality and intelligence—have been examined through many lenses in L2 education contexts: emotional intelligence (EI), cognitive abilities, investment in language study, willingness to communicate, etc. Evidence has been found that personality and cognitive or intelligence-related variables influence how students approach L2 tasks, how they deal with communicative challenges, and how efficiently they process linguistic input (e.g., Sasaki, 1993; Wilson & Lynn, 1990).

Although previous research has advanced parts of the understanding of the role of personality and intelligence in L2 learning (Anglim et al., 2022; Wang & Liu, 2023), many gaps remain. First, studies have either focused on personality (e.g., Oz, 2015; Dewaele, 2019) or intelligence (e.g., Taheri et al., 2019; Slevc & Miyake, 2006), and very few have attempted to explore the terrain of both domains at once. Second, comprehensive frameworks for investigating multiple dimensions of intelligence (cognitive, emotional, cultural) and personality have not yet been explicitly integrated into L2 research, although researchers call for widening the scope for constructs such as trait EI (Dewaele et al., 2008; Ożańska-Ponikwia, 2011, 2015). Third, the learning environment has been further complicated by new technological interventions, including intelligent personal assistants (IPAs) such as Amazon Alexa, Google Assistant, and Apple Siri. These tools serve as real-time communication practice resources that are supposed to take L2 learning into account by engaging cognitive and emotional factors such as working memory, motivation, and willingness to communicate (Tai, 2022; Tai & Chen, 2022). However, there is still a lack of attention to the interplay between personality and intelligence in the context of the use of IPAs in language learning.

### 1.1. Personality, Emotional Intelligence, and L2 Success

Personality characteristics have been studied extensively in education research through many frameworks, ranging from the Big Five to the HEXACO model (Furnham & Monsen, 2009; Meyer et al., 2023; Quílez-Robres et al., 2023). Within specific L2 contexts, Wilson and Lynn (1990) examined the relationships between personality traits (psychoticism, neuroticism, and extraversion), cognitive abilities, and second language (L2) learning outcomes among approximately 1200 children in the Irish Republic learning Irish as the second language, finding that while personality traits were not strongly related to cognitive abilities, there were low but positive correlations between attitudes toward foreign language learning and both Irish language test scores and certain cognitive measures, such as English spelling and verbal fluency. To this effect, however, attitudes and personality turned out to mediate language achievement. Sasaki (1993) indicated that intelligence- and personality-related factors certainly affect L2 proficiency; nonetheless, they have unique roles.

Different frameworks of viewing intelligence refer to emotional intelligence (EI) as independent constructions and include it as an interrelated dimension of personality (Dewaele et al., 2018; Pishghadam & Sahebjam, 2012). Considering its meanings, some authors treat EI as a form of intelligence influencing L2 learning (Chen et al., 2021; Oz et al., 2015); other researchers do not consider it except as a personality-related cause, especially via trait EI models (Dewaele et al., 2008; Ożańska-Ponikwia, 2011, 2015). Research findings show that EI contributes to L2 success as it moderates anxiety (Li et al., 2021; Rahman & Tomy, 2023), promotes engagement (McEown et al., 2023; Alamer & Alrabai, 2024), and leads to better proficiency in speaking (Ożańska-Ponikwia et al., 2020). Therefore, EI is essential because it provides the double perspective that seems relevant to understand the complexity of its role in language learning and the need for further exploration of its interfaces with personality traits.

### 1.2. Intelligence, Cognitive Abilities, and L2 Performance

Whereas EI and personality have garnered strong attention in L2 contexts, researchers have examined a wide array of general and specific cognitive abilities, including reasoning, verbal intelligence, working memory, and even implicitly theorized writing intelligence (Waller & Papi, 2017). L2 performance as understood in this review includes proficiency in speaking, writing, listening, and reading, measurable with standardized tests or observational studies. In contrast, L2 success is a broader term that includes performance, and, in addition, is concerned with longer-term learning outputs, motivation, adaptability, and overall efficiency in language acquisition. Sasaki (1993) showed that second language proficiency involves both a general SLP factor (G-SLP) and a general cognitive factor (G-COG). More than half of the variance in G-SLP was explained by variables other than G-COG, confirming the complexity underlying L2 achievement. Other studies emphasize intelligence in its varied forms—beyond IQ—such as cultural intelligence (Lin et al., 2012; Poort et al., 2021) and multiple intelligences (Akbari & Hosseini, 2008; Haley, 2004; Hasbullah et al., 2023).

For instance, Oz et al. (2015) found that emotional intelligence contributed significantly to foreign language learning quality, suggesting that intelligence cannot be reduced to a single dimension if we aim to understand the wide variance in L2 outcomes. In the same vein, Taheri et al. (2019) explored links between cognitive intelligence, emotional intelligence, and L2 achievement, showing that multiple facets of cognitive and noncognitive intelligence simultaneously shape L2 success. More straightforward relationships, such as the correlation between “traditional” intelligence measures and L2 skills, have been reported by Wilson and Lynn (1990) (in the context of Irish language learners) and by Slevc and Miyake (2006) (in investigating the role of music aptitude and general intelligence in L2 phonology). Moreover, recent concerns about how intelligence interplays with personality are highlighted by large-scale meta-analyses (Anglim et al., 2022; Stanek & Ones, 2023), thereby laying a foundation for an integrated investigation of personality, multiple intelligences, and second language performance.

### 1.3. Role of Technology and IPAs in L2 Learning

With the increasing integration of technology in education, IPAs have emerged as potential facilitators of L2 learning. They provide immediate, authentic, and interactive communication practice (Moussalli & Cardoso, 2019; Wu et al., 2020) and are possible catalysts for language learning outside formal settings (Yang et al., 2022). Nevertheless, the use of IPAs in learning creates challenges that include difficulties concerning comprehension and production for those learners who have higher degrees of foreign accent or lower levels of proficiency in general (Moussalli & Cardoso, 2019). However, studies indicate that the personality traits of learners and their cognitive abilities can actually impact the benefits that may be derived from IPAs. Willingness to communicate may moderate them in their ability to effectively use these tools (Oz, 2015), while anxiety levels, according to Rahman and Tomy (2023), challenge the way learners choose to use these tools. Working memory is an example of a cognitive ability that affects how IPA-generated input is understood and retained (Dizon, 2021).

The shift to online learning and technology-mediated instruction—accelerated by the COVID-19 pandemic—has further emphasized the role of digital tools in L2 acquisition, as seen in studies about emotional terra incognita (He et al., 2024; Resnik et al., 2021; Wang et al., 2024), teacher well-being (Dewaele et al., 2018), and engagement (Alamer & Alrabai, 2024; Yu et al., 2022). The rapid shift to online contexts raises questions about how intelligence and personality might predict learners’ behaviors, self-regulation, and ultimate achievement in newly digitized L2 environments (Liu et al., 2023; Reza, 2020). Research on technology tools, whether IPAs (Tai, 2022) or entire online ecosystems (Rastegar & Kermani, 2015), underscores the continued need to conceptualize intelligence and personality as complex, interdependent dimensions shaping learning trajectories.

### 1.4. Integrating Personality, Intelligence, and L2 Outcomes

Drawing a synthesis, the current study found that personality and intelligence remain relevant predictors of success, though hardly the same across this broad spectrum, which is especially true in terms of language learning. Earlier research made use of extraversion, neuroticism, or IQ per se (Wilson & Lynn, 1990; Sasaki, 1993) alone to measure their relation to language-learning ability; on the other hand, advancing the idea of measuring language attributes, new studies boast high emotional and cultural intelligence (Lin et al., 2012; Soldatova & Geer, 2013), and advanced personality frameworks are also developed (Dewaele, 2019; Meyer et al., 2023). Additional complicating factors include multiple intelligences (Akbari & Hosseini, 2008; Behjat, 2012; Haley, 2004; Hasbullah et al., 2023) and grit or mindset (Khajavy & Aghaee, 2022; Eren & Rakıcıoğlu-Söylemez, 2020).

There have been increasing associations between emotional and interpersonal dimensions of intelligence (EI, trait EI, cultural intelligence, etc.) and L2 enjoyment, reduced anxiety, and more effective oral communication (Dewaele et al., 2008; Shao et al., 2013; Wang & Liu, 2023). For example, greater emotional regulation capacities (Shafiee Rad & Hashemian, 2022) and greater well-being (Shafiee Rad & Hashemian, 2022; Reza, 2020) facilitate L2 performance by fostering more positive learner mindsets, reducing inhibition, and increasing motivation (Thao et al., 2023; Herrera et al., 2020; Yu et al., 2023). The advantage brought by personality traits, such as extraversion or openness (Furnham & Monsen, 2009; Stanek & Ones, 2023), can act in combination with domain-specific intelligence factors (Taheri et al., 2019; Sasaki, 1993) to contribute to greater inter-learner variability in L2 success.

Finally, a systematic analysis of personality and intelligence in relation to L2 outcomes is relatively minimal. While some meta-analyses have considered personality and intelligence in general (Anglim et al., 2022; Stanek & Ones, 2023), or EI and L2 achievement in particular (Wang & Liu, 2023; Vashisht et al., 2021), there is noticeably no study researching these constructs in the context of second or foreign language learning. For that reason, this paper is intended to fill that void by employing the range of empirical studies reviewed earlier, from classic experiments on personality and L2 attainment (Wilson & Lynn, 1990) to contemporary findings on EI, grit, cultural intelligence, IPAs, and beyond (Chen et al., 2021; Tai, 2022), in a systematic synthesis of how personality and intelligence jointly predict L2 learning success. While IPAs are not inherent psychological constructs, their integration into learning environments necessitates consideration of how they mediate the effects of personality and intelligence on L2 success. The phrase “and beyond” was originally intended to capture emerging variables such as domain-specific trait emotional intelligence (L2-TEI) and the evolving role of digital learning tools. However, to maintain conceptual clarity, we refined the focus to psychological constructs while acknowledging the mediating role of technology in the L2 learning processes. As a result, this systematic review seeks to answer the following questions: (1) To what extent do personality and intelligence jointly predict second or foreign language (L2) learning success? (2) How do different dimensions of personality and intelligence (e.g., cognitive, emotional, cultural) interact in shaping L2 achievement?

## 2. Materials and Methods

**Figure 1:**
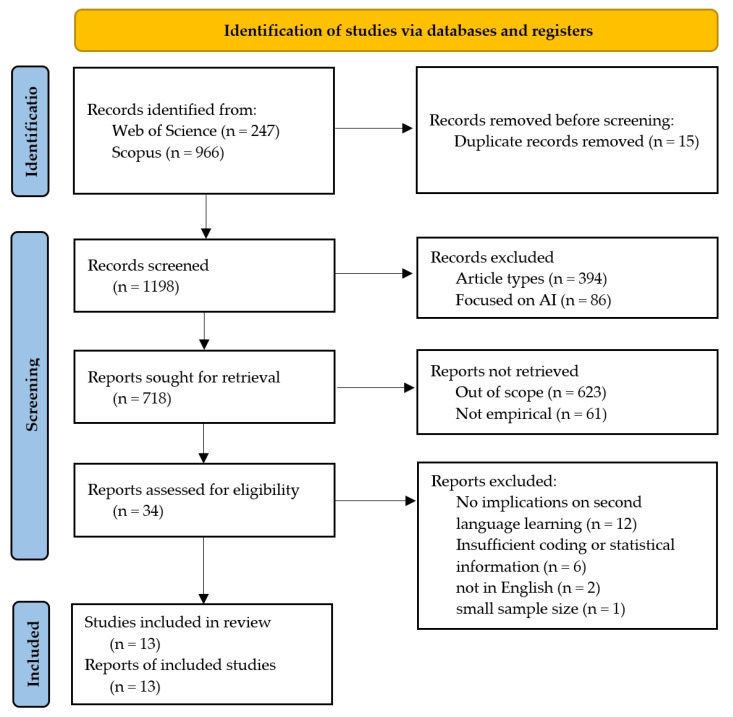
A PRISMA flow diagram of the literature selection process.

This research was a systematic review of studies following the PRISMA guidelines (Page et al., 2021) to examine the relationship between personality, intelligence, and second language learning success. It was not registered in a publicly accessible registry, and a formal review protocol was not prepared. The studies were identified through a broad search on 28 December 2024 of two major databases, Web of Science and Scopus. These databases were chosen due to their extensive indexing of peer-reviewed research, particularly in the fields of psychology, linguistics, and education, which are directly relevant to the study of personality, intelligence, and second language learning. Web of Science covers high-impact journals with citation tracking systems enabling large-scale searches for important studies. Scopus offers, amongst its many international multidisciplinary sources, extensive new and emerging research from various educational contexts. These combined databases guarantee a comprehensive yet proportional literature collection for this systematic review (see Figure 1). The search strategy was designed to encompass studies published between 2000 and 2024. The following keywords and their variations were used: “personality”, “intelligence”, “emotional intelligence”, “cognitive abilities”, “second language learning”, “foreign language acquisition”, “language proficiency”, “grit”, “motivation”, and “language learning strategies”. Boolean operators (AND, OR) were applied to refine the search and ensure comprehensive coverage of the relevant literature. The searches yielded 1213 records in total (Web of Science: 247; Scopus: 966).

Fifteen duplicate records were removed, leaving 1198 unique records for screening. The initial title and abstract screening excluded 480 records based on the following criteria: article type not suited to this analysis (e.g., opinion pieces, book chapters, non-peer-reviewed sources) (*n* = 394) and studies primarily focused on artificial intelligence rather than cognitive or personality factors in L2 learning (*n* = 86). A full-text review was conducted on the remaining 718 records. At that stage, 684 records had to be excluded. Of these, 623 were clearly out of scope since they did not directly examine the personality, intelligence, and second language learning relationship, and instead were focused on the general cognitive development, other forms of academic performance not related to language, or applications of artificial intelligence that do not involve human personality traits. Additionally, 61 were not empirical, as they comprised theoretical papers, reviews of the literature, or conceptual discussions devoid of original data or statistical analysis. Following this rigorous screening, 34 studies were assessed for eligibility based on strict inclusion criteria, ensuring that only empirical research with direct relevance to personality, intelligence, and L2 learning was considered. At that stage, 21 studies were further excluded because they had no direct relevance to second language learning (*n* = 12); lacked coding or statistical information (*n* = 6); were in a language other than English (*n* = 2); or had a very small sample size (*n* = 1). This resulted in a total of 13 studies satisfying all the inclusion criteria and included in the final systematic review. Two independent reviewers extracted data from each included report, working independently to ensure accuracy, with no automation tools used and no additional processes for obtaining or confirming data from study investigators. These 13 studies represent a diverse range of methodologies, sample populations, and L2 learning contexts, ensuring a well-rounded and reliable synthesis of the existing research.

Data were sought for the target outcomes: (1) second language learning success, measured by proficiency or achievement in the second language (e.g., test scores, fluency assessments); (2) personality traits, operationalized using accepted constructs such as the Big Five personality traits; and (3) intelligence, as measured by tests of cognitive abilities and related assessments. Data compatible with each of the outcome domains were extracted for the measure, timepoints, and analyses reported in the studies, with priority given, where multiple measures or timepoints were reported, to the primary or broadest outcome measure within each domain. Priority was assigned based on what was stated in the study objectives or the relevance of the measure to the review questions. The author also searched for the characteristics of participants (e.g., age, gender, educational background, native language, any previous experience in learning a second language), characteristics of interventions (type and duration of instruction or exposure, mode of pedagogy, language that was learned), study features (year of publication, study design, sample size, geographic location), and sources of funding (e.g., existence and source of funding). Given missing or vague information, the missing information was inferred using contextual details provided in the study (e.g., age may be inferred from expressions such as “university students”). However, studies where intervention or outcome information could be considered critical were excluded from the analysis when this information was insufficient.

Though the included studies were referenced, the Cochrane Risk of Bias Tool assessed the risk of bias in terms of other domains, namely, selection bias, performance bias, detection bias, and reporting bias. Two independent reviewers independently assessed each study to maintain objectivity and consistency. No automated software was used in the process of assessing the risk of bias. Any disagreements between the reviewers were settled by mutual discussion; if they were unable to settle, a third reviewer was called to assist in the discussion. The following measures of effect were employed: for continuous outcomes (for example, test scores or proficiency measures)—mean difference (MD); for dichotomous outcomes (such as successes versus failures in achieving a predefined level of proficiency)—risk ratio (RR). Where appropriate, standardized mean differences (SMD) were employed to adjust for discrepancies in measurement scales across studies. To determine the eligibility of each synthesis, each study’s characteristics (e.g., intervention type, duration, population, outcome) were tabulated and compared against the predefined criteria for each synthesis group as given in the protocol. If a study’s intervention, population, and outcome matched one or more of the eventual comparisons to be drawn, it was included in a specific synthesis. The exercise was performed by two independent reviewers, and any discrepancies were resolved by discussion or by consulting a third reviewer. Studies that did not meet the eligibility criteria for any of the syntheses were excluded from further analysis. As part of data preparation, missing summary statistics (e.g., means and standard deviations) were calculated from available data, medians were converted into means where appropriate, and outcome measurements were standardized across different scales. Synthesis was not conducted for studies with insufficient data. Moreover, the author considered the geographical and cultural contexts of the studies, as well as the types of interventions and learning environments (e.g., classroom-based versus technology-mediated learning).

## 3. Results

The review consisted of thirteen studies that spanned different methodologies, populations, and contexts for a broad understanding of the relation between personality and intelligence and success in second language learning. Geographically, the studies were conducted in different countries and involved learners of different ages, proficiency levels, and target languages. As there were only a few studies included in the review (*n* = 13), and, therefore, a significant amount of heterogeneity existed among the studies, sensitivity testing was deemed futile. Hence, no formal methods of assessing publication bias risk or any funnel plots were used to check for bias due to missing results. Data coded concerning the studies included sample size characteristics, personality and intelligence measures, definitions and evaluations of second language learning success, and statistical analyses used. Certainty (or confidence) in the body of evidence for each outcome was not explicitly rated with tools such as GRADE because the review was chiefly concerned with synthesizing findings, not assessing the strength of the evidence. The detailed information on these studies is listed in Table 1.

Essentially, this systematic review collects the results from thirteen different studies investigating how personality and EI, cognitive abilities, and L2 learning success are related to each other. The studies also demonstrate the belief that individual differences have a significant impact on learner performance, engagement, and overall experience in second language contexts. Among them, emotional intelligence and personality traits appear to play the most important roles in shaping multiple dimensions of language learning, including speaking skills, learning strategies, and affective states. Other constructs such as personal intelligence and working memory, as well as conditions such as mindset and grit, have been found not to be well-integrated with personality and emotional intelligence in predicting overall outcomes for language. Thus, the second language learning process appears complex and fluid in that a single intervention is not sufficient to meet the requirements of different learners.

### 3.1. Emotional Intelligence and Second Language Learning

Various emotions have been discussed regarding the success of L2 learning since its early days. Alamer and Alrabai (2024) confirmed the importance of the L2-TEI scale and established that global TEI and its subdomains of well-being and sociability explain 73% of the learner engagement variance. In quite the same vein, Chen and Zhang (2020) showed that global TEI as well as some of its dimensions such as well-being and emotionality were reliable predictors of listening, speaking, and English proficiency, suggesting that EI is also significant in terms of L2 performance in speaking, reading, listening, and writing.

EI was also associated with teacher-related factors. According to Dobakhti et al. (2022), EFL instructors’ EI affected their teacher immunity more than other psychological problems, such as neuroticism and openness. As an example, Sobhanmanesh (2022) found that EI accounted well for flow states in EFL teachers, while major activities such as reading and teaching provided an optimal experience. Ożańska-Ponikwia (2011, 2015) reported that students’ EI traits such as empathy, emotional perception, and regulation significantly affected their emotional expression in L2 use through self-report. These findings bring about a complex understanding of EI as well as its role in learners’ perceptions and management of emotions during the use of the second language.

### 3.2. Personality Traits and Second Language Learning

Personality traits were another major contributor to L2 learning success that influenced learners and teachers alike. These were followed by openness to experience, conscientiousness, and extraversion in various studies, which seemed to ensure better L2 outcomes across all studies. For instance, Sobhanmanesh (2022) reported conscientiousness and openness as the strongest predictors of flow states among EFL teachers. The study by Taheri et al. (2019) identified agreeableness and extraversion as the key influences on emotional expression in L2 use. Gu and Sharil (2023) also observed that extroverts preferred group-based strategies, whereas introverts preferred independent learning approaches.

Nowbakht and Fazilatfar (2019) reported that extroversion significantly enhanced speaking ability by accounting for 53.6% of its variance, along with working memory and interpersonal intelligence. This provides further evidence of the role of personality in highly interactive L2 tasks. Research by Ewa Krautz (2021) and Ożańska-Ponikwia (2011) investigated how personality traits, such as extraversion and openness, relate to bilinguals’ self-reported impressions of being “a different person” when switching languages. These findings indicate that personality influences not only performance, but also learners’ subjective experience in L2 contexts.

### 3.3. Cognitive and Psychological Constructs in L2 Learning

Some of the cognitive intelligence, working memory, and psychological factors such as grit, mindset, and flow states also emerged as important factors in defining L2 outcomes. Nowbakht and Fazilatfar (2019) reported that working memory significantly predicted verbal intelligence and was one of the strongest contributors to speaking ability. Taheri et al. (2019) found that cognitive intelligence (IQ) and some subdomains of emotional intelligence, such as interpersonal relationships, were highly related to L2 achievement.

Fan et al. (2024) highlighted how the interactions of growth language mindset (GLMS) and grit influence L2 engagement and burnout. The relationship between perseverance of effort (POE) and grit was stronger compared to the consistency of interest (COI), which denotes that it is a sustained effort that is the significant determinant of success in L2 learning. Sobhanmanesh (2022) showed that flow states, or a state of deep absorption either in teaching or reading activities, are influenced by personality and EI. These findings thus indicate that fostering flow states may enhance not only teaching quality, but also teacher satisfaction.

### 3.4. Learning Strategies and Styles

In addition to investigating how individual differences affect learning strategies and preferences, Gu and Sharil (2023) found that personality types had a significant influence on learning strategies as well. Sensing learners tend to concentrate on practical applications, whereas intuitive learners prefer abstract concepts. Thus, it may be possible to optimize learning outcomes by matching strategies with personality types. Taheri et al. (2019) demonstrated that both EI and IQ were significantly related to the use of cognitive, compensation, and social strategies employed by learners, thus linking intelligence with strategic flexibility when executing L2 tasks.

Vongkrahchang and Chinwonno (2016) showed how PI training enhanced goal-setting and strategy use for Thai university students and indicated the promising potential of focused interventions in improving discrete skills. Cognitive intelligence and working memory, along with other psychological constructs such as grit, mindset, and states of flow, play crucial roles in shaping L2 outcomes.

### 3.5. Affective and Cultural Factors

**Figure 2:**
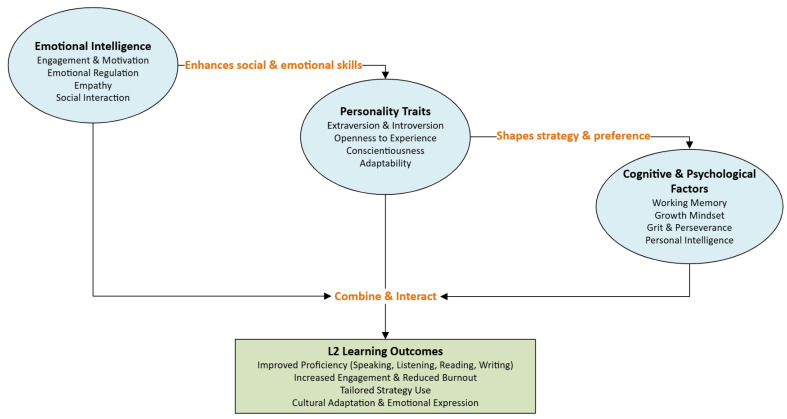
Summary of the results.

Mood, affect, and culture also play a significant role in shaping the learning process of a foreign language. According to Miller and Godfroid (2020), mood states (being positive, negative, or neutral) can even interact with personality traits to influence the acquisition process of a foreign language. It was observed that participants who were able to manage stress effectively performed well on the language learning task, indicating that mood and personality are interrelated. According to the results from the studies analyzed, the feeling of being a “different person” among bilinguals was examined in conjunction with personality traits, emotional intelligence, and cultural immersion as linked factors, suggesting that L2 learning involves more than just cognitive aspects, but also emotional and cultural dimensions. Some factors have been found to correlate with emotional intelligence, personality traits, cognitive and psychological factors, as well as independent variables, all contributing to L2 learning outcomes (see Figure 2).

In L2 contexts, emotional intelligence induces learner engagement, motivation, and social adaptation by integrating components such as well-being, sociability, emotional regulation, and empathy. Personality traits, such as extraversion, openness to experience, and conscientiousness, influence learners’ preferences for strategies and tasks, with extraverts excelling in group interactions and introverts favoring independent learning. Cognitive and psychological factors, including working memory, growth mindset, grit, and personal intelligence, provide the cognitive and motivational tools necessary for language acquisition, supporting learners in retaining information and persevering through challenges. These factors interact in meaningful ways: EI and personality traits collectively govern social and emotional dimensions of learning; personality shapes how cognitive strategies are employed; and EI supports stress management and focus, amplifying the effects of cognitive abilities. Together, these interconnected elements contribute to improved L2 proficiency, sustained engagement, tailored learning strategies, and greater cultural and emotional adaptation.

## 4. Discussion

### 4.1. Personality and Emotional Intelligence as Core Predictors of L2 Outcomes

In the reviewed studies, Alamer and Alrabai (2024), Chen and Zhang (2020), Dobakhti et al. (2022), Ożańska-Ponikwia (2011, 2015), Sobhanmanesh (2022), and Taheri et al. (2019) presented evidence consistent with the notion that emotional intelligence—frequently measured in subdomains such as well-being, empathy, self-control, and sociability—is strongly related to various outcomes of L2 learning, namely, engagement, achievement, self-perceived changes in behavior, and performance in specific language skills (listening, speaking, reading, and writing). In the same vein, personality traits, and especially extraversion, openness to experience, and conscientiousness, emerged as good predictors of performance in L2 (Nowbakht & Fazilatfar, 2019; Taheri et al., 2019), even teacher immunity (Dobakhti et al., 2022) and teacher flow (Sobhanmanesh, 2022). These findings correspond to large meta-analyses by Anglim et al. (2022), Meyer et al. (2023), and Stanek and Ones (2023), which show that openness, extraversion, and conscientiousness are strong correlates of academic achievement and intelligence-related variables.

### 4.2. Personality and Emotional Intelligence as Core Predictors of L2 Success

Research has established, as shown in many of the studies reviewed, that emotional intelligence (EI) constitutes a significant factor in L2 learning, in conjunction with personality traits (Ożańska-Ponikwia, 2011, 2015; Chen & Zhang, 2020). Well-being, sociability, and self-control have been cited time and again as EI components that correlate with increased motivation in learning English as a foreign language. This implies that personality traits such as extraversion and conscientiousness prove to be either enhancers or debuffs to the likely benefits of EI for learning a second language.

Correlative studies also focus on the traits that students share concerning how they regulate their emotions in the L2 learning process. Such personality traits commonly include neuroticism, which is shown to raise anxiety levels, causing detrimental effects on the performance of language students (Rahman & Tomy, 2023). Compared to those who are not, learners with good emotional intelligence and high self-regulation can manage anxiety and increase in public motivation, resulting in the possibility of successful outcomes. The results bring forward a new idea for an integrative approach that entails personality and emotional intelligence for understanding the success of L2 learners.

### 4.3. Self-Regulation, Engagement, and Affective Dimensions in L2 Learning and Teaching

The significance of one’s idiosyncrasy has repeatedly been the most common subject in all of the studies when it comes to bringing motivation, resilience, and deeper learning experiences to the fore. Grit and mindset (Fan et al., 2024) lower burnout and increase engagement, supporting the idea that perseverance, positivity, and resilience are mediators of anxiety’s negative effect on L2 performance (Chen et al., 2021; Dewaele et al., 2018; Xu et al., 2022).

Other variables pertain to teachers, too. According to the studies on teacher immunity (Dobakhti et al., 2022) and teacher flow (Sobhanmanesh, 2022), a teacher’s emotional and personality resources tend to affect the quality of teaching and thus student outcomes. This is consistent with the general fact that affective variables such as emotional intelligence and personality at the teacher level, in turn, affect the dynamics inside the classroom (Miller & Godfroid, 2020; Stanek & Ones, 2023).

### 4.4. Theoretical Integration and Future Directions

Theoretically, together, these articles reinforce the multiplicity of constructs shaping L2 outcomes, namely, the Big Five personality dimensions, emotional intelligence domains, grit, mindset, working memory, and personal intelligence. This observation is consistent with Sakai and Kikuchi’s (2009) demand for multifaceted models of L2 attainment and Sasaki (1993)’s assertion that general cognitive ability accounts for only part of the variance in L2 performance.

Such findings further underscore the importance of context or domain-specific constructs such as L2-TEI (Alamer & Alrabai, 2024) or personal intelligence (Vongkrahchang & Chinwonno, 2016) for advancing our thinking beyond general measures of intelligence and personality. Further, such findings can be replicated with different language populations or developmental levels and in various settings such as immersion versus classroom and face-to-face versus online learning.

Finally, these 13 studies did not focus on technological contexts, such as IPAs or virtual classes. However, given the importance of personality, EI, and intelligence in the success of language learning, there is a high probability that these variables will be examined within these learner–machine interactions (cf. Dizon, 2017; Tai, 2022). Such an expansion would reflect the current trend in the field toward hybrid or fully online L2 education, prompted by the integration of today’s technology into education.

**Figure 3:**
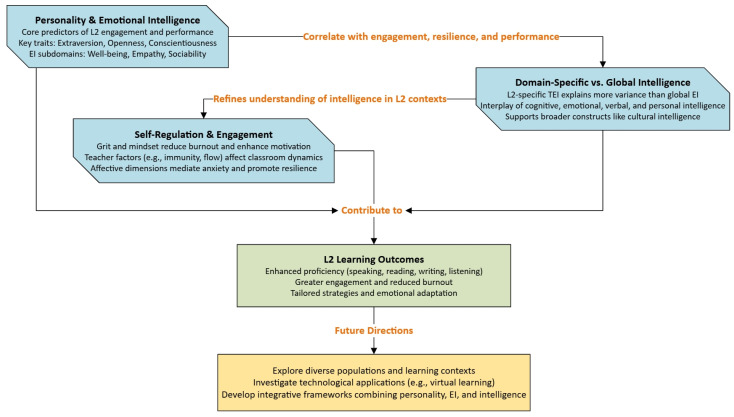
Conceptual framework of the key predictors, outcomes, and future directions in L2 learning.

Thus, taken collectively, these 13 studies indicate that personality and intelligence—and most specifically emotional intelligence—serve as vital explanatory constructs for understanding why and how learners differ in L2 achievement, L2 engagement, and emotional experience (see Figure 3). Different traits or subdomains (e.g., conscientiousness, extraversion, well-being, empathy) offer a strong predictive power for language performance, resilience, and identity shifts. Such a synthesis also shows domain-specific approaches (e.g., L2-TEI, personal intelligence) to further explore the complex interplay of individual differences in L2 contexts. Such revelations closely resemble the broader research arena where personality dimensions and intelligence measures have been associated with academic attainment (Meyer et al., 2023; Stanek & Ones, 2023), emotional–behavioral outcomes (Quílez-Robres et al., 2023; Vashisht et al., 2021), and second language achievements (Dewaele et al., 2008; Taheri et al., 2019). Looking forward, integrated systems evaluating the synergy of trait EI with general intelligence, specific forms of intelligence (such as cultural intelligence and personal intelligence), and personality in conjunction with contextual variables (such as the use of IPAs, virtual learning environments) will provide critical building blocks for intervention design, teacher training modules, and personalized language learning pathways.

### 4.5. Limitations and Implications

In this review, the evidence considered is subject to some problems. Many of the studies examined here provided useful insights into the influence of personality and intelligence on the second language proficiency, but some used small or specific sample sizes, limiting their application to a broad range of situations. Moreover, many concentrated on specific traits (e.g., extraversion, openness) instead of looking at these considerations from the perspective of a wide range of personality and intelligence factors, something that probably precludes a deeper understanding of these seemingly complex relations. Furthermore, their utility is limited due to the wide use of cross-sectional designs, which do not provide evidence to determine how such learner characteristics are related to language outcomes across time and, perhaps, how they vary from one cultural context to another. In carrying out this systematic review, the author relied mainly on database searches in Web of Science and Scopus and excluded works published outside of these indices. Consequently, some relevant studies may have been missed, influencing the overall patterns and conclusions. Further, although screening was conducted at all levels, constructs such as personality, intelligence, and emotional intelligence are bound to be subjectively coded. The different choices made in coding may have further constrained the interpretation of the results.

This review implies that individual differences in personality and emotional intelligence must be considered when teaching and learning a second language. Language instructors and education officials could build intervention programs that promote emotional regulation or adaptive skills for teachers and students alike, thereby fostering engagement and learning outcomes. In the future, researchers should use and benefit from measurement approaches that have more standardization and robustness in addition to consistent analytical frameworks. In this case, previously validated instrument scales (such as TEI Questionnaire–SF for emotional intelligence, or the Big Five for personality) should be favored, whereby reliability can be ensured in replicating findings across different contexts. Another suggestion is to use longitudinal and mixed-methods designs rather than mostly cross-sectional data to track personality, intelligence, and L2 outcomes continuously. Advanced analytical techniques such as multilevel modeling or latent profile analysis would also allow for greater specificity in distinguishing which learner subgroups respond differently to particular interventions or exhibit distinct trajectories of L2 development. Through this systematic and multipronged approach, the field would increase the understanding of how both domain-general and domain-specific measures of intelligence interact with personality attributes in determining second language success.

## 5. Conclusions

As indicated by this review, personality traits and intelligence types play a significant role in the second language (L2) learning success. Evidence across the studies suggests that extraversion, openness, conscientiousness, and certain forms of emotional intelligence, empathy, self-control, and sociability are significant predictors of L2 achievement, learner engagement, and resilience. Cognitive resources such as working memory, grit, and personal intelligence were observed to interact with affective and behavioral variables, demonstrating the results of an organized combination of emotionally grounded motivation and cognitive considerations in language acquisition. The findings in this area have continued to accumulate, but have been limited by small sample sizes, limited populations, and often cross-sectional designs, which make it difficult to examine the evolution of traits and intelligence over time when these designs are not used. As the present evidence suggests, recognizing and developing the appropriate personality and intelligence factors can be accomplished through a combination of specific interventions, teacher training, and technology-mediated learning. This will lead to the enhancement of learners, as well as their ability to perform in the language. To fill some of the research gaps and to put these individual differences within the context of a world in which second language education is becoming increasingly diverse, further research is required to apply domain-specific emotional intelligence measures and integrate advanced technologies, such as intelligent personal assistants.

## Figures and Tables

**Table 1:** Detailed information on the included studies.

Author	Region	Source	Sample	Participants	Variables	Operationalization	Statistical Methods
Alamer and Alrabai (2024)	Saudi Arabia	*Acta Psychologica*	415	Language learners	Language domain-specific trait emotional intelligence, language engagement	L2-TEI scale	Confirmatory factor analysis (CFA), exploratory structural equation modeling (ESEM), bifactor CFA, and bifactor ESEM
Chen and Zhang (2020)	China	*Journal of Multilingual and Multicultural Development*	72	Chinese postgraduate EFL learners	Trait emotional intelligence (EI), well-being, self-control, emotionality, sociability, overall English performance	TEI Questionnaire—Short Form (TEIQue—SF), English proficiency test	Regression analysis
Dobakhti et al. (2022)	Iran	*Teaching English Language*	129	EFL teachers	Personality traits, EI, teacher immunity	Emotional intelligence questionnaire, personality scale, teacher immunity questionnaire	Multiple regression analysis
Ewa Krautz (2021)	Germany	*Journal of Multilingual and Multicultural Development*	452	L1 German–L2 English speakers	Feeling like a different person, psychological constructs of tolerance of ambiguity, L2 proficiency	Online questionnaire	Binary logistic regression (GLM model)
Fan et al. (2024)	China	*System*	868	First-year high-school EFL learners	L2 grit, L2 burnout, L2 engagement	Language Mindset Inventory, L2-grit scale, Maslach Burnout Inventory—Student Survey, L2 engagement scale	Structural equation modelling (SEM)
Gu and Sharil (2023)	Malaysia	*Educational Administration: Theory and Practice*	250	Non-English major undergraduate students	MBTI, language learning strategies	Myers–Briggs Type Indicator (MBTI), online questionnaire	Structural equation model (SEM) with partial least squares (PLS)
Miller and Godfroid (2020)	US	*Studies in Second Language Acquisition*	120	Unknown	Mood states, openness, intuition, emotional intelligence, foreign language anxiety, impulsivity	Individual differences questionnaires	Between-subjects and within-subjects statistical techniques
Nowbakht and Fazilatfar (2019)	Iran	*Journal of Asia TEFL*	60	Upper-intermediate EFL students	working memory, introversion vs. extroversion Personality types, verbal and interpersonal intelligences in EFL speaking ability	WM task, intelligence and personality test, oral interview	Multiple regression analysis
Ożańska-Ponikwia (2011)	Poland	*International Journal of Bilingual Education and Bilingualism*	102	Polish–English bilinguals and Polish L2 users of English	OCEAN personality test, EI, the notion of “feeling different” while using a foreign language	OCEAN personality test, TEI questionnaire	Stepwise regression analysis
Ożańska-Ponikwia (2015)	Poland	*International Journal of Bilingual Education and Bilingualism*	102	Polish L2 users of English	Extraversion, agreeableness, conscientiousness, emotional stability/neuroticism, EI, self-reported emotional expression in L2	L2 questionnaire, TEI questionnaire, Big Five broad domains personality test	Correlation analysis
Sobhanmanesh (2022)	Canada	*Frontiers in Psychology*	75	EFL teachers	Big Five personality, EI, flow states	Experience sampling forms, emotional quotient inventory, revised NEO personality inventory	Correlation analysis, *t*-test, multiple regression analysis
Taheri et al. (2019)	Iran	*Cogent Education*	188	Iranian EFL learners	Cognitive intelligence, emotional intelligence, learning styles, language learning strategies, L2 achievement	Raven’s progressive matrices, BarOn emotional quotient inventory, Kolb’s learning style inventory, strategy inventory for language learning	Correlation analysis, one-way ANOVA
Vongkrahchang and Chinwonno (2016)	Thailand	*Kasetsart Journal of Social Sciences*	39	Undergraduates majoring in English	Personal intelligence, goal setting, monitoring, evaluation strategy	Personal intelligence reading instruction (PIRI), personal intelligence inventory (PI inventory), classroom observation form	Mixed methods

## Data Availability

No data were created for this article.

## References

[B1-behavsci-15-00428] Akbari R., Hosseini K. (2008). Multiple intelligences and language learning strategies: Investigating possible relations. System.

[B2-behavsci-15-00428] Alamer A., Alrabai F. (2024). The second language trait emotional intelligence (L2-TEI) scale and its relation to language engagement. Acta Psychologica.

[B3-behavsci-15-00428] Anglim J., Dunlop P. D., Wee S., Horwood S., Wood J. K., Marty A. (2022). Personality and intelligence: A meta-analysis. Psychological Bulletin.

[B4-behavsci-15-00428] Behjat F. (2012). Interpersonal and intrapersonal intelligences: Do they really work in foreign-language learning?. Procedia—Social and Behavioral Sciences.

[B5-behavsci-15-00428] Chen Z., Zhang P. (2020). Trait emotional intelligence and second language performance: A case study of Chinese EFL learners. Journal of Multilingual and Multicultural Development.

[B6-behavsci-15-00428] Chen Z., Zhang P., Lin Y., Li Y. (2021). Interactions of trait emotional intelligence, foreign language anxiety, and foreign language enjoyment in the foreign language speaking classroom. Journal of Multilingual and Multicultural Development.

[B7-behavsci-15-00428] Dewaele J.-M. (2019). Multilingualism and trait emotional intelligence: An exploratory investigation. International Journal of Multilingualism.

[B8-behavsci-15-00428] Dewaele J.-M., Gkonou C., Mercer S. (2018). Do ESL/EFL teachers’ emotional intelligence, teaching experience, proficiency and gender affect their classroom practice?. Emotions in second language teaching.

[B9-behavsci-15-00428] Dewaele J.-M., Petrides K. V., Furnham A. (2008). Effects of trait emotional intelligence and sociobiographical variables on communicative anxiety and foreign language anxiety among adult multilinguals: A review and empirical investigation. Language Learning.

[B10-behavsci-15-00428] Dizon G. (2017). using intelligent personal assistants for second language learning: A case study of alexa. TESOL Journal.

[B11-behavsci-15-00428] Dizon G. (2021). Affordances and constraints of intelligent personal assistants for second-language learning. RELC Journal.

[B12-behavsci-15-00428] Dobakhti L., Zohrabi M., Masoudi S. (2022). Scrutinizing the affective predictors of teacher immunity in foreign language classrooms. Teaching English Language.

[B13-behavsci-15-00428] Eren A., Rakıcıoğlu-Söylemez A. (2020). Language mindsets, perceived instrumentality, engagement and graded performance in English as a foreign language students. Language Teaching Research.

[B14-behavsci-15-00428] Ewa Krautz A. (2021). Tolerance of ambiguity, need for cognitive closure and feeling like a different person when speaking different languages. Journal of Multilingual and Multicultural Development.

[B15-behavsci-15-00428] Fan N., Yang C., Kong F., Zhang Y. (2024). Low-to mid-level high school first-year EFL learners’ growth language mindset, grit, burnout, and engagement: Using serial mediation models to explore their relationships. System.

[B16-behavsci-15-00428] Furnham A., Monsen J. (2009). Personality traits and intelligence predict academic school grades. Learning and Individual Differences.

[B17-behavsci-15-00428] Gu Y., Sharil W. N. E. H. (2023). Study on the effect of personality type on the language learning strategies of non-English major students through MBTI test. Eğitim Yönetimi.

[B18-behavsci-15-00428] Haley M. H. (2004). Learner-Centered Instruction and the theory of multiple intelligences with second language learners. Teachers College Record.

[B19-behavsci-15-00428] Hasbullah, Wahidah N., Nanning (2023). Integrating multiple intelligence learning approach to upgrade students’ English writing skills. International Journal of Language Education.

[B20-behavsci-15-00428] He R., Xu W., Dong D., Yu Z. (2024). A meta-analysis of the effect of interactive technologies on language education. International Journal of Adult Education and Technology.

[B21-behavsci-15-00428] Herrera L., Al-Lal M., Mohamed L. (2020). Academic achievement, self-concept, personality and emotional intelligence in primary education. Analysis by gender and cultural group. Frontiers in Psychology.

[B22-behavsci-15-00428] Khajavy G. H., Aghaee E. (2022). The contribution of grit, emotions and personal bests to foreign language learning. Journal of Multilingual and Multicultural Development.

[B23-behavsci-15-00428] Li C., Huang J., Li B. (2021). The predictive effects of classroom environment and trait emotional intelligence on foreign language enjoyment and anxiety. System.

[B24-behavsci-15-00428] Lin Y., Chen A. S., Song Y. (2012). Does your intelligence help to survive in a foreign jungle? The effects of cultural intelligence and emotional intelligence on cross-cultural adjustment. International Journal of Intercultural Relations.

[B25-behavsci-15-00428] Liu L., Fathi J., Allahveysi S. P., Kamran K. (2023). A model of teachers’ growth mindset, teaching enjoyment, work engagement, and teacher grit among EFL teachers. Frontiers in Psychology.

[B26-behavsci-15-00428] McEown K., McEown M. S., Oga-Baldwin W. L. Q. (2023). The role of trait emotional intelligence in predicting academic stress, burnout, and engagement in Japanese second language learners. Current Psychology.

[B27-behavsci-15-00428] Meyer J., Jansen T., Hübner N., Lüdtke O. (2023). Disentangling the association between the big five personality traits and student achievement: Meta-analytic evidence on the role of domain specificity and achievement measures. Educational Psychology Review.

[B28-behavsci-15-00428] Miller Z. F., Godfroid A. (2020). Emotions in incidental language learning. Studies in Second Language Acquisition.

[B29-behavsci-15-00428] Moussalli S., Cardoso W. (2019). Intelligent personal assistants: Can they understand and be understood by accented L2 learners?. Computer Assisted Language Learning.

[B30-behavsci-15-00428] Nowbakht M., Fazilatfar A. M. (2019). The effects of working memory, intelligence and personality on English learners’ speaking ability. The Journal of Asia TEFL.

[B31-behavsci-15-00428] Oz H. (2015). Emotional intelligence as a predictor of L2 communication. Procedia—Social and Behavioral Sciences.

[B32-behavsci-15-00428] Oz H., Demirezen M., Pourfeiz J. (2015). Emotional intelligence and attitudes towards foreign language learning: Pursuit of relevance and implications. Procedia—Social and Behavioral Sciences.

[B33-behavsci-15-00428] Ożańska-Ponikwia K. (2011). What has personality and emotional intelligence to do with “feeling different” while using a foreign language?. International Journal of Bilingual Education and Bilingualism.

[B34-behavsci-15-00428] Ożańska-Ponikwia K. (2015). Are women more emotionally skilled when it comes to expression of emotions in the foreign language? Gender, emotional intelligence and personality traits in relation to emotional expression in the L2. International Journal of Bilingual Education and Bilingualism.

[B35-behavsci-15-00428] Ożańska-Ponikwia K., Piechurska-Kuciel E., Skałacka K. (2020). Emotional intelligence as a mediator in the relationship between neuroticism and L2 achievement. Applied Linguistics Review.

[B36-behavsci-15-00428] Page M. J., McKenzie J. E., Bossuyt P. M., Boutron I., Hoffmann T. C., Mulrow C. D., Shamseer L., Tetzlaff J. M., Akl E. A., Brennan S. E., Chou R., Glanville J., Grimshaw J. M., Hróbjartsson A., Lalu M. M., Li T., Loder E. W., Mayo-Wilson E., McDonald S., McGuinness L. A. (2021). The PRISMA 2020 statement: An updated guideline for reporting systematic reviews. British Medical Journal.

[B37-behavsci-15-00428] Pishghadam R., Sahebjam S. (2012). Personality and emotional intelligence in teacher burnout. The Spanish Journal of Psychology.

[B38-behavsci-15-00428] Poort I., Jansen E., Hofman A. (2021). Cultural intelligence and openness to experiences pave the way for cognitive engagement in intercultural group work. Journal of Studies in International Education.

[B39-behavsci-15-00428] Quílez-Robres A., Usán P., Lozano-Blasco R., Salavera C. (2023). Emotional intelligence and academic performance: A systematic review and meta-analysis. Thinking Skills and Creativity.

[B40-behavsci-15-00428] Rahman A., Tomy P. (2023). Intelligent personal assistant—An interlocutor to mollify foreign language speaking anxiety. Interactive Learning Environments.

[B41-behavsci-15-00428] Rastegar M., Kermani E. M. (2015). Emotional intelligence, tolerance of ambiguity, and language learning strategies use of EFL learners: A study of relations. Cumhuriyet Üniversitesi Fen Edebiyat Fakültesi Fen Bilimleri Dergisi.

[B42-behavsci-15-00428] Resnik P., Moskowitz S., Panicacci A. (2021). Language learning in crisis mode: The connection between LX grit, trait emotional intelligence and learner emotions. The Journal for the Psychology of Language Learning.

[B43-behavsci-15-00428] Reza A. (2020). The effectiveness of flipped learning in a testing university classroom: Students’ perceptions, the role of personality traits, and successful intelligence. Applied Research on English Language.

[B44-behavsci-15-00428] Sakai H., Kikuchi K. (2009). An analysis of demotivators in the EFL classroom. System.

[B45-behavsci-15-00428] Sasaki M. (1993). Relationships among second language proficiency, foreign language aptitude, and intelligence: A structural equation modeling approach. Language Learning.

[B46-behavsci-15-00428] Shafiee Rad H., Hashemian M. (2022). Role of hedonic and eudaimonic well-being in second language learners’ trait emotional intelligence and emotion regulation. European Journal of Psychology of Education.

[B47-behavsci-15-00428] Shao K., Yu W., Ji Z. (2013). An exploration of Chinese EFL students’ emotional intelligence and foreign language anxiety. The Modern Language Journal.

[B48-behavsci-15-00428] Slevc L. R., Miyake A. (2006). Individual differences in second-language proficiency. Psychological Science.

[B49-behavsci-15-00428] Sobhanmanesh A. (2022). English as a foreign language teacher flow: How do personality and emotional intelligence factor in?. Frontiers in Psychology.

[B50-behavsci-15-00428] Soldatova G., Geer M. (2013). “Glocal” identity, cultural intelligence and language fluency. Procedia—Social and Behavioral Sciences.

[B51-behavsci-15-00428] Stanek K. C., Ones D. S. (2023). Meta-analytic relations between personality and cognitive ability. Psychological and Cognitive Sciences.

[B52-behavsci-15-00428] Taheri H., Sadighi F., Bagheri M. S., Bavali M., Khajavi Y. (2019). EFL learners’ L2 achievement and its relationship with cognitive intelligence, emotional intelligence, learning styles, and language learning strategies. Cogent Education.

[B53-behavsci-15-00428] Tai T.-Y. (2022). Effects of intelligent personal assistants on EFL learners’ oral proficiency outside the classroom. Computer Assisted Language Learning.

[B54-behavsci-15-00428] Tai T.-Y., Chen H. H.-J. (2022). The impact of intelligent personal assistants on adolescent EFL learners’ listening comprehension. Computer Assisted Language Learning.

[B55-behavsci-15-00428] Thao L. T., Thuy P. T., Thi N. A., Yen P. H., Thu H. T. A., Tra N. H. (2023). Impacts of emotional intelligence on second language acquisition: English-major students’ perspectives. SAGE Open.

[B56-behavsci-15-00428] Vashisht S., Kaushal P., Vashisht R. (2021). Emotional intelligence, personality variables and career adaptability: A systematic review and meta-analysis. Vision: The Journal of Business Perspective.

[B57-behavsci-15-00428] Vongkrahchang S., Chinwonno A. (2016). Effects of personal intelligence reading instruction on personal intelligence profiles of Thai university students. Kasetsart Journal of Social Sciences.

[B58-behavsci-15-00428] Waller L., Papi M. (2017). Motivation and feedback: How implicit theories of intelligence predict L2 writers’ motivation and feedback orientation. Journal of Second Language Writing.

[B59-behavsci-15-00428] Wang Y., Liu F. (2023). Emotional intelligence and second/foreign language achievement: A meta-analytic review. Language Teaching Research.

[B60-behavsci-15-00428] Wang Y., Xu W., Guan K., Zhao J., Wu P. (2024). English teachers’ post-pandemic motivation in macau’s higher education system. Theory and Practice in Language Studies.

[B61-behavsci-15-00428] Wilson R. G., Lynn R. (1990). Personality, intelligence components and foreign language attainment. Educational Psychology.

[B62-behavsci-15-00428] Wu Y., Rough D., Bleakley A., Edwards J., Cooney O., Doyle P. R., Clark L., Cowan B. R. (2020). See what I’m saying? Comparing intelligent personal assistant use for native and non-native language speakers. 22nd International Conference on Human-Computer Interaction with Mobile Devices and Services.

[B63-behavsci-15-00428] Xu W., Zhang H., Sukjairungwattana P., Wang T. (2022). The roles of motivation, anxiety and learning strategies in online Chinese learning among Thai learners of Chinese as a foreign language. Frontiers in Psychology.

[B64-behavsci-15-00428] Yang C. T.-Y., Lai S.-L., Chen H. H.-J. (2022). The impact of intelligent personal assistants on learners’ autonomous learning of second language listening and speaking. Interactive Learning Environments.

[B65-behavsci-15-00428] Yu Z., Sukjairungwattana P., Xu W. (2022). Effects of serious games on student engagement, motivation, learning strategies, cognition, and enjoyment. International Journal of Adult Education and Technology.

[B66-behavsci-15-00428] Yu Z., Xu W., Sukjairungwattana P. (2023). Motivation, learning strategies, and outcomes in mobile English language learning. The Asia-Pacific Education Researcher.

